# Arterial hyperoxia and in-hospital mortality after resuscitation from cardiac arrest

**DOI:** 10.1186/cc10090

**Published:** 2011-03-08

**Authors:** Rinaldo Bellomo, Michael Bailey, Glenn M Eastwood, Alistair Nichol, David Pilcher, Graeme K Hart, Michael C Reade, Moritoki Egi, D James Cooper

**Affiliations:** 1 Australian and New Zealand Intensive Care Research Centre, School of Public Health and Preventive Medicine, Monash University, 5 Commercial Road, Prahran, Melbourne, Victoria 3181, Australia; 2 Australia New Zealand Intensive Care Society (ANZICS) Clinical Outcomes and Resource Evaluation (CORE) Centre, 10 Ievers Terrace, Carlton, Melbourne, Victoria 3053, Australia; 3 Department of Intensive Care, Austin Hospital, 145 Studley Road, Heidelberg, Melbourne, Victoria 3084, Australia; 4 Department of Anesthesiology and Resuscitology, Okayama University Medical School, 5-1 Shikata-Cho 2-Chome, Okayama 700-8558, Okayama, Japan

## Abstract

**Introduction:**

Hyperoxia has recently been reported as an independent risk factor for mortality in patients resuscitated from cardiac arrest. We examined the independent relationship between hyperoxia and outcomes in such patients.

**Methods:**

We divided patients resuscitated from nontraumatic cardiac arrest from 125 intensive care units (ICUs) into three groups according to worst PaO_2 _level or alveolar-arterial O_2 _gradient in the first 24 hours after admission. We defined 'hyperoxia' as PaO_2 _of 300 mmHg or greater, 'hypoxia/poor O_2 _transfer' as either PaO_2 _< 60 mmHg or ratio of PaO_2 _to fraction of inspired oxygen (FiO_2 _) < 300, 'normoxia' as any value between hypoxia and hyperoxia and 'isolated hypoxemia' as PaO_2 _< 60 mmHg regardless of FiO_2_. Mortality at hospital discharge was the main outcome measure.

**Results:**

Of 12,108 total patients, 1,285 (10.6%) had hyperoxia, 8,904 (73.5%) had hypoxia/poor O_2 _transfer, 1,919 (15.9%) had normoxia and 1,168 (9.7%) had isolated hypoxemia (PaO_2 _< 60 mmHg). The hyperoxia group had higher mortality (754 (59%) of 1,285 patients; 95% confidence interval (95% CI), 56% to 61%) than the normoxia group (911 (47%) of 1,919 patients; 95% CI, 45% to 50%) with a proportional difference of 11% (95% CI, 8% to 15%), but not higher than the hypoxia group (5,303 (60%) of 8,904 patients; 95% CI, 59% to 61%). In a multivariable model controlling for some potential confounders, including illness severity, hyperoxia had an odds ratio for hospital death of 1.2 (95% CI, 1.1 to 1.6). However, once we applied Cox proportional hazards modelling of survival, sensitivity analyses using deciles of hypoxemia, time period matching and hyperoxia defined as PaO_2 _> 400 mmHg, hyperoxia had no independent association with mortality. Importantly, after adjustment for FiO_2 _and the relevant covariates, PaO_2 _was no longer predictive of hospital mortality (*P *= 0.21).

**Conclusions:**

Among patients admitted to the ICU after cardiac arrest, hyperoxia did not have a robust or consistently reproducible association with mortality. We urge caution in implementing policies of deliberate decreases in FiO_2 _in these patients.

## Introduction

The majority of patients who experience cardiac arrest die at the time of the event [1,2]. Even after response to resuscitation efforts and survival to intensive care unit (ICU) admission, such patients have a short-term mortality of approximately 60% [1,2]. These dismal outcomes suggest the need for strategies to attenuate postresuscitation injury. Such injury is currently mostly attributed to cerebral, myocardial and global ischemia-reperfusion injury [3]. Accordingly, postresuscitation therapy has focused on finding ways to diminish the intensity and consequences of ischemia-reperfusion injury.

The rapid application of therapeutic hypothermia can modify the outcomes of patients after resuscitation from cardiac arrest [4,5]. The success associated with this intervention suggests that other aspects of patient care, which may influence the course of reperfusion injury, should also be logical targets for therapeutic manipulation.

In pursuit of potential therapeutic targets, investigators from the Emergency Medicine Shock Research Network (EMShockNet) recently explored the association between hyperoxia and in-hospital outcome in a retrospective, multicentre study [6]. They found that hyperoxia occurred in almost one-fifth of patients, that patients with hyperoxia had greater in-hospital mortality than patients with normoxia or hypoxia and that, after controlling for some confounders, hyperoxia carried a clear independent association with mortality (odds ratio (OR), 1.8). Unfortunately, these investigators used only the first set of arterial blood gases in the ICU to assess oxygenation, excluded close to 30% of patients because of lack of arterial blood gas data and did not adjust for standard illness severity scores. Their conclusion that hyperoxia is a robust predictor of mortality in patients after resuscitation form cardiac arrest was therefore potentially affected by selection bias and by insufficient adjustment for major confounders. Thus, their results are of uncertain significance and require confirmation.

The Australian and New Zealand (ANZ) Adult Patient Database (ANZ-APD) is a high-quality database [7] of all admissions to most Australian and New Zealand ICUs. Patients admitted after resuscitation from nontraumatic cardiac arrest are coded as such. The database records contain Acute Physiology and Chronic Health Evaluation II (APACHE II) and APACHE III scores as well as demographic, diagnostic and outcome factors [8]. Given the potential clinical importance of hyperoxia following cardiac arrest, we used this larger and more detailed database specifically to confirm or refute the initial findings of the EMShockNet investigators [6].

## Materials and methods

We extracted data from the ANZ Intensive Care Society (ANZICS)-APD. We used exactly same inclusion criteria as the EMShockNet investigators [6] for patients admitted at a participating centre between 2000 and 2009 after resuscitation from an out-of-hospital or in-hospital cardiac arrest. We excluded readmissions and patients for whom arterial blood gas analysis or vital status at discharge was not available. The ANZICS Centre for Outcomes and Resource Evaluation (CORE) Management Committee granted us access to the data in accordance with standing protocols. Data were collected under the Quality Assurance Legislation of the Commonwealth of Australia (Part VC Health Insurance Act 1973, Commonwealth of Australia) with government support and funding. Each hospital gives ethics approval and allows the data to be used for appropriate research, which is governed by the ANZICS CORE terms of reference and waives the need for informed consent.

### Data collection for oxygen values

All arterial blood gases during the first 24 hours of ICU admission were collected and entered into a standardized data collection system which automatically selects the appropriate high and low simultaneous fraction of inspired oxygen (FiO_2_) and partial pressure of arterial oxygen (PaO_2_) measurements and deletes other oxygenation data. Using the APACHE II and III methodology for intubated patients with FiO_2 _≥0.5, the PaO_2 _associated with the arterial blood gas with the highest alveolar-arterial (A-a) gradient is selected as the index of worst oxygenation. For nonintubated patients or intubated patients with FiO_2 _< 0.5, the lowest arterial blood gas PaO_2 _level is recorded. The ratio of PaO_2 _to FiO_2 _(P/F ratio) is also used as an index of illness severity.

### Data extraction

We recorded the sizes, types and locations of the hospitals. At the patient level, we extracted the following variables: demographics, comorbidities according to APACHE II and III classifications, hospital and ICU admission source, intubation, treatment limitation, year of admission, physiological and arterial blood gas parameters over the first 24 hours in the ICU, vital status at hospital discharge, hospital discharge destination and an APACHE III risk of death score [8]. As a marker of severity of illness independent of arterial oxygenation, we calculated an adjusted APACHE III index of illness severity (AP3no-ox), in which the oxygen component of the APACHE III scoring system was removed.

### Statistical analyses

All analyses were performed using SAS version 9.2 (SAS Institute Inc., Cary, NC, USA). Continuous data are presented as means ± standard deviations or as medians with interquartile ranges (IQRs), depending on the underlying data distribution. Categorical data are reported as proportions. We categorized oxygenation levels into the same three groups as in the EMShockNet study [6], defined by the worst PaO_2 _and P/F ratio obtained in the first 24 hours of ICU admission. Thus, we divided patients into three groups according to worst PaO_2 _or A-a O_2 _gradient in the first 24 hours after admission. We defined 'hyperoxia' as a PaO_2 _300 mmHg or greater, 'hypoxia/poor O_2 _transfer' as either PaO_2 _< 60 mmHg or a P/F ratio <300, 'normoxia' as any value between hypoxia and hyperoxia and 'isolated hypoxemia' as PaO_2 _< 60 mmHg regardless of FiO_2 _level.

The primary outcome measures were in-hospital mortality and survival time, which are reported as ORs (95% confidence interval (95% CI)) or hazard ratios (HRs) (95% CI), respectively. To determine functional recovery, we also considered discharge to home as a secondary outcome. We compared outcomes between groups using the χ^2 ^test with the Bonferroni correction. We conducted multivariate analysis using logistic regression for mortality and Cox proportional hazards regression for survival time, with models constructed using both stepwise selection and backwards elimination procedures. To increase robustness and model validity, we used a *P *value of 0.01 for variable inclusion. We applied several models to the statistical analysis of the independent relationship between oxygenation and patient outcome. We constructed an initial model for mortality in accordance with the EMShockNet model (see Additional file 1, Statistical appendix, Model 1) [8]. We then applied a second model to improve discriminatory power using AP3no-ox as a marker of severity (see Additional file 1, Statistical appendix, Model cluster 2). Finally, we conducted further sensitivity analysis inclusive of propensity analysis [9], Cox proportional hazards modelling, testing of different cutoff points for hyperoxia, analysis of subgroups contemporaneous with the EMShockNet cohort and assessment of PaO_2 _according to deciles (Additional file 1, Statistical appendix, Model cluster 3).

As our database contained only the worst recorded oxygenation in the first 24 hours after ICU admission, we explored its relationship with that of the first PaO_2 _measurement after ICU admission (as in the EMShockNet study) and the mean oxygenation on ICU admission days 1, 2 and 3 by selecting 100 of the database patients and obtaining additional data from all of their hospital arterial blood gas records during their ICU stay (see Additional file 1, Statistical appendix, Model cluster 3).

### Statistical power considerations

The proportion of living patients with hyperoxia (PaO_2 _> 400) was 5% (*n *= 280). Comprising 5,140 patients who lived and 6,968 patients who died, this study had 93% power to detect a change of 1.5% (5% versus 6.5%) in the proportion of patients with hyperoxia (PaO_2 _> 400) with a two-sided *P *value of 0.05.

There were 625 patients in the data set with hyperoxia (PaO_2 _> 400). Comprising 11,483 patients without hyperoxia, this study had 90% power to detect a difference in mortality of 7% (55% versus 62%) between groups with a two-sided *P *value of 0.05. Given an observed difference of 14% (55% versus 69%) in the EMShockNet study between hyperoxic patients (PaO_2 _> 400) and nonhyperoxic patients, we felt that this study was adequately powered to detect a relationship between mortality and PaO_2 _> 400. In our study, the mortality rate in the hyperoxia group (PaO_2 _> 400) was only 0.5% higher than that in the nonhyperoxia group (54.7% versus 55.2%, *P *= 0.22).

There were 531 hyperoxia survivors (PaO_2 _> 300). With 4,609 nonhyperoxia survivors, this study had 80% power to detect a difference between groups of 6% (64% versus 58%) regarding the proportion discharged to home with a two-sided *P *value of 0.05. Given an observed difference of 6% (38% versus 44%) in the EMShockNet study between hyperoxic survivors (PaO_2 _> 300) discharged to home and discharged nonhyperoxic survivors, we again felt that this study was adequately powered to detect a relationship between hyperoxia and discharge to home. In our study, there was no observed difference in the proportion of patients who were discharged to home between the hyperoxia and nonhyperoxia groups.

## Results

There were 12,806 patients who met the study inclusion criteria. Of these, 698 (5.4%) were excluded: 222 (1.7%) had missing arterial blood gas data, 382 (3.0%) had missing hospital mortality data and 94 (0.7%) were ICU readmissions. The remaining 12,108 patients were drawn from among 125 contributing ICUs. The median number of cardiac arrest cases per hospital was 42 (IQR, 13-148). Baseline characteristics for all groups are given in Tables 1 and 2.

**Table 1:** Baseline characteristics of the study patients^a^

Patient characteristics	All patients (*N *= 12,108)	Hypoxia/poor O_2 _exchange (*n *= 8,904)	Normoxia (*n *= 1,919)	Hyperoxia (*n *= 1,285)
Mean age, yr (±SD)	64 (16)	64 (16)	62 (18)	65 (17)
Male sex, *n *(%)	7,802 (64)	5,778 (65)	1,228 (64)	796 (62)
Indigenous Australians, *n *(%)	515 (5)	388 (5)	74 (4)	53 (4)
Hospital admission source from home, *n *(%)	8,175 (68)	5,986 (67)	1,273 (66)	916 (71)
Acute renal failure, *n *(%)	2,368 (20)	1,916 (22)	237 (12)	215 (17)
Chronic comorbidities, *n *(%)				
Cardiovascular disease, *n *(%)	2,395 (20)	1,821 (20)	357 (19)	217 (17)
Liver disease, *n *(%)	194 (2)	158 (2)	17 (1)	19 (1)
Renal disease, *n *(%)	668 (6)	488 (5)	100 (5)	80 (6)
Respiratory disease, *n *(%)	1,044 (9)	831 (9)	102 (5)	111 (9)
Cirrhosis, *n *(%)	195 (2)	158 (2)	18 (1)	19 (1)
Hepatic failure, *n *(%)	70 (1)	52 (1)	10 (1)	8 (1)
Immune suppression, *n *(%)	329 (3)	243 (3)	41 (2)	45 (4)
Cancer, *n *(%)	413 (3)	320 (4)	48 (3)	45 (4)
Markers of severity				
Median APACHE III risk of death (IQR)	66% (36 to 84)	69% (40 to 86)	50% (20 to 73)	66% (36 to 84)
Median APACHE III risk of death (no oxygen)^b ^(IQR)	58% (27 to 79)	60% (29 to 80)	47% (18 to 71)	58% (29 to 80)
ICU admission source, *n *(%)				
Emergency department	5,756 (48)	4,123 (46)	1,035 (54)	598 (47)
Operating theatre	1,261 (10)	925 (10)	217 (11)	119 (9)
Other hospital	1,958 (16)	1,445 (16)	319 (17)	194 (15)
Ward	3,113 (26)	2,397 (27)	344 (18)	372 (29)
Treatment limitation^c^	562 (5)	429 (5)	68 (4)	65 (5)

^a^APACHE III, Acute Illness Severity and Chronic Health Evaluation III; IQR, interquartile range; ICU, intensive care unit; ^b^APACHE III risk of death with oxygen component removed from APACHE III score; ^c^treatment limitation order or coded for palliative care.

**Table 2:** Baseline characteristics of the study hospitals

Hospital characteristics, *n *(%)	All patients (*N *= 12,108)	Hypoxia/poor O_2 _exchange (*n *= 8,904)	Normoxia (*n *= 1,919)	Hyperoxia (*n *= 1,285)
Hospital size^a^				
Small to medium (≤300 beds)	2,475 (20)	1,813 (20)	361 (19)	301 (23)
Large (301 to 500 beds)	5,277 (44)	3,906 (44)	843 (44)	528 (41)
Extra large (>500 beds)	4,356 (36)	3,185 (36)	715 (37)	456 (35)
Hospital type and location				
Metropolitan community	2,670 (22)	1,988 (22)	437 (23)	245 (19)
Private	787 (6)	573 (6)	89 (5)	125 (10)
Rural	1,279 (11)	939 (11)	205 (11)	135 (11)
Tertiary academic	7,372 (61)	5,404 (61)	1,188 (62)	780 (61)

^a^Defined according to Halpern *et al. *[30].

The average age of patients was 64 years (SD ± 16), and 64% (7,802) were male. A total of 8,175 patients (68%) were at home prior to hospital admission and 5,756 patients (48%) were admitted to the ICU directly from the Emergency Department. One-third (3,978) of the patients had preexisting chronic comorbidities. The median APACHE III risk of death was 66% (IQR, 36%-84%). Most patients (8,904, 73.5%) had 'hypoxia/poor O_2 _transfer', while 1,285 (10.6%) were hyperoxic and 1,919 (15.9%) were normoxic. Isolated hypoxemia (PaO_2 _< 60 mmHg) was present in 1,168 patients (9.7%).

There were no significant differences in the measured physiological data between the three main oxygenation groups (Table 3). Patients had a median lowest temperature of 34.9°C, and in 33% of the patients, this value was below 34.0°C. The median ICU length of stay for survivors from ICU admission to hospital discharge was 3.8 days (IQR, 2.0 to 7.1), and for nonsurvivors it was 1.5 days (IQR, 0.5 to 3.3). The median length of hospital stay for survivors was 14.9 days (IQR, 8.2 to 27.2), and for nonsurvivors it was 3.4 days (IQR, 1.5 to 8.1).

**Table 3:** Abnormal vital signs in the first 24 hours in intensive care unit and interventions

Vital signs (means ± SD)	All patients (*N *= 12,108)	Hypoxia/poor O_2 _exchange (*n *= 8,904)	Normoxia (*n *= 1,919)	Hyperoxia (*n *= 1,285)
Highest body temperature	37.1°C (1.5)	37.1°C (1.5)	37.1°C (1.4)	37.1°C (1.5)
Lowest body temperature	34.9°C (1.7)	34.9°C (1.7)	34.8°C (1.8)	34.7°C (1.7)
Highest heart rate, beats/min	108 (28)	109 (28)	104 (26)	108 (28)
Highest respiratory rate, breaths/min	22.0 (9.0)	22.2 (9.0)	21.4 (9.2)	21.4 (9.0)
Lowest systolic blood pressure, mmHg	88.6 (25.1)	87.3 (25.0)	94.1 (22.8)	88.9 (27.2)
Lowest mean arterial pressure, mmHg	62.3 (16.0)	61.5 (15.8)	66.2 (14.5)	62.5 (18.0)
Lowest glucose level first 24 hours	6.9 (3.9)	6.9 (4.0)	6.4 (3.1)	6.9 (3.6)
Body temperature, *n *(%)				
Highest temperature <34°C	860 (7)	639 (7)	90 (5)	131 (10)
Lowest temperature <34°C	4031 (33)	2918 (33)	659 (34)	454 (35)

Overall, 6,968 patients (58%) died in the hospital (Table 4). Mortality was significantly lower (*P *< 0.0001) in the normoxia group than in either the hyperoxia group or the hypoxia/poor O_2 _transfer group. It was highest, however, in patients with 'isolated hypoxemia' (812 (70%) of 1,168 patients, *P *< 0.0001). The proportion of patients discharged directly to home was significantly higher in the normoxia group than in the other groups. The lowest rate of discharge to home was in patients with isolated hypoxemia (222 (19%) of 1,168 patients, *P *< 0.0001). Overall, 65% of survivors were discharged directly to home.

**Table 4:** Outcomes of study patients

Patient outcomes	All patients (*N *= 12,108)	Hypoxia/poor O_2 _exchange (*n *= 8,904)	Normoxia (*n *= 1,919)	Hyperoxia (*n *= 1,285)
In-hospital mortality^a^, *n *(%) (95% CI)	6,968 (58) (57 to 58)	5,303 (60) (59 to 61)	911 (47) (45 to 50)	754 (59) (56 to 61)
Discharge destination for survivors, *n*	5,140	3,601	1,008	531
Home ^a^, *n *(%) (95% CI)	3,341 (28) (27 to 28)	2,350 (26) (25 to 27)	649 (34) (32 to 36)	342 (27) (24 to 29)
Rehabilitation facility	655 (5)	447 (5)	118 (6)	90 (7)
Transfer to another hospital	1,144 (9)	804 (9)	241 (13)	99 (8)

^a^*P *< 0.0001 for comparisons of normoxia with hyperoxia and normoxia with hypoxia in patients discharged to home.

When the EMShockNet statistical model was replicated, 12 risk factors were significantly associated with in-hospital mortality (Table 5). Data were well fitted by the model (Hosmer-Lemeshow goodness-of-fit test, *P *= 0.71), and the area under the curve (AUC) was 0.72. Hypoxia/poor O_2 _transfer or hyperoxia were significantly associated with an increased risk of mortality in comparison to normoxia (OR 1.4 (95% CI, 1.3 to 1.6), *P *< 0.0001, and OR 1.5 (95% CI, 1.3 to 1.8), *P *< 0.0001, respectively). Once illness severity was added to the model (Table 6) (Additional file 1, Statistical appendix, Model cluster 2), the magnitude of the effect size was markedly lower than in the original EMShockNet model (hypoxia versus normoxia: OR 1.2 (95% CI, 1.1 to 1.4), *P *= 0.002; hyperoxia versus normoxia: OR 1.2 (95% CI, 1.0 to 1.5), *P *= 0.04). This APACHE-based model showed improved discriminatory power in comparison to the EMShockNet model (AUC 0.79 when AP3no-ox was applied in isolation versus AUC 0.81 when AP3no-ox was applied in combination with other variables listed in Table 6). Data were well fitted by the model (Hosmer-Lemeshow goodness-of-fit test, *P *= 0.42).

**Table 5:** Multiple logistic regression model with in-hospital mortality as dependent variable using EMShockNet model variables^a^

Variable	OR (95%CI)	*P *value
Acute renal failure	3.3 (2.9 to 3.7)	<0.0001
Hypotension in first 24 hours^b^	1.9 (1.7 to 2.0)	<0.0001
Age, decile	1.1 (1.1 to 1.1)	<0.0001
Emergency department origin	1.6 (1.4 to 1.7)	<0.0001
High heart rate^c^	1.5 (1.3 to 1.6)	<0.0001
Hypoxia/poor O_2 _exchange versus normoxia	1.4 (1.3 to 1.6)	<0.0001
Hyperoxia versus normoxia	1.5 (1.3 to 1.8)	<0.0001
Cancer	2.0 (1.5 to 2.5)	<0.0001
Cirrhosis	2.2 (1.5 to 3.1)	<0.0001
Female sex	1.2 (1.1 to 1.3)	<0.0001
Chronic renal	1.4 (1.1 to 1.6)	0.001
Chronic respiratory disease	1.3 (1.1 to 1.5)	0.002
Hepatic failure	2.7 (1.3 to 5.9)	0.01

^a^EMShockNet, Emergency Medicine Shock Research Network; OR, odds ratio; 95% CI, 95% confidence interval. The following variables (OR (95% CI), *P *value) were removed from the model for nonsignificance (*P *< 0.01): immunosuppression (1.3 (1.0 to 1.7), *P *= 0.04), indigenous status (1.2 (0.9 to 1.5), *P *= 0.13), chronic cardiovascular disease (1.1 (0.9 to 1.2), *P *= 0.34), chronic liver disease (0.6 (1.0 to 3.3), *P *= 0.56) and hospital source prior to admission being from home (1.0 (0.9 to 1.1), *P *= 0.61). ^b^Defined as any systolic blood pressure of less than 90 mmHg in the first 24 hours; ^c^indicates highest value for first 24 hours in the intensive care unit (1 = exceeds median and 0 = median or lower).

**Table 6:** Multiple regression models for in-hospital mortality and survival time using an APACHE III-based marker of severity^a^

Variable	Hospital mortality OR (95% CI)	*P *value	Time to death HR (95% CI)	*P *value
AP3no-ox^b^	1.5 (1.5 to 1.6)	<0.0001	1.2 (1.2 to 1.2)	<0.0001
Treatment limitation^c^	5.3 (3.8 to 7.2)	<0.0001	1.7 (1.5 to 1.8)	<0.0001
Year of admission	0.9 (0.9 to 0.9)	<0.0001	0.97 (0.96 to 0.98)	<0.0001
Lowest glucose in first 24 hours	1.1 (1.1 to 1.1)	<0.0001	1.02 (1.02 to 1.03)	<0.0001
Hospital admission from home	1.3 (1.1 to 1.4)	0.0002	1.1 (1.0 to 1.1)	0.02
Hypoxia/poor O_2 _exchange versus normoxia	1.2 (1.1 to 1.4)	0.002	1.1 (1.0 to 1.2)	0.01
Hyperoxia versus normoxia	1.2 (1.0 to 1.5)	0.04	1.1 (1.0 to 1.2)	0.20

^a^APACHE III, Acute Illness Severity and Chronic Health Evaluation III; OR, odds ratio; 95% CI, 95% confidence interval; HR, hazard ratio; AP3no-ox, APACHE III score with oxygenation component removed; ^b^APACHE III risk of death with oxygen component removed from calculation algorithm; ^c^treatment limitation order or palliative care coded for the patient. Indigenous status was removed from both models for nonsignificance (*P *< 0.01): (OR 1.3 (95% CI, 1.0 to 1.8), *P *= 0.04), (HR 1.1 (95% CI, 0.9 to 1.1) *P *= 0.89).

Propensity analysis (see Additional file 1, Statistical appendix, Model cluster 3) did not alter this risk or the significance of hyperoxia. However, when the secondary outcome of discharge to home was considered, oxygenation status was no longer a statistically significant predictor (*P *= 0.64). Using a Cox proportional hazards regression model, we found both hyperoxia and hypoxia/poor O_2 _transfer to increase the hazard of death in comparison to the normoxia group (HR 1.3 (95% CI, 1.1 to 1.4), *P *< 0.001, and HR 1.3 (95% CI, 1.2 to 1.4), *P *< 0.0001, respectively). After adjustment for the covariates described in Additional file 1, Statistical appendix, Model cluster 2, however, oxygenation status was no longer statistically significant (hyperoxia: OR 1.1 (95% CI, 1.0 to 1.2), *P *= 0.20; hypoxia: OR 1.1 (95% CI, 1.0 to 1.2), *P *= 0.01) (Table 6).

When a PaO_2 _of 200 mmHg or greater was used to define hyperoxia, after adjustment (Additional file 1, Statistical appendix, Model cluster 2), oxygenation status was a statistically significant predictor of outcome (*P *= 0.002) (hyperoxia: OR 1.3 (95% CI, 1.1 to 1.5), *P *= 0.01; hypoxia: OR 1.3 (95% CI, 1.1 to 1.5), *P *= 0.001). When a PaO_2 _of 400 mmHg or greater was used in sensitivity analysis after adjustment, however (Additional file 1, Statistical appendix, Model cluster 2), oxygenation status was no longer statistically significant (*P *= 0.06) (hyperoxia: OR 1.0 (95% CI, 0.8 to 1.2), *P *= 0.71; hypoxia: OR 1.1 (95% CI, 1.0 to 1.3), *P *= 0.04).

**Figure 1:**
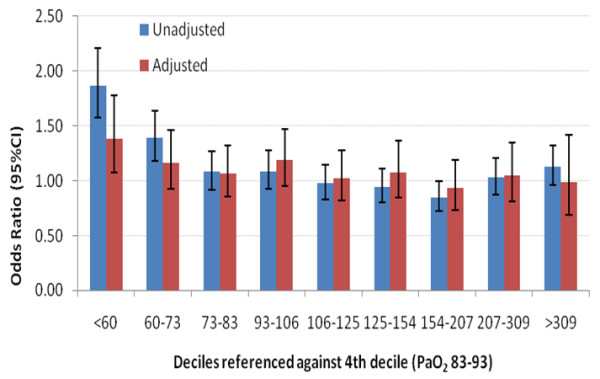
**Odds ratios for hospital mortality by deciles of PaO_2_**. Odds ratios for hospital mortality with partial pressure of arterial oxygen (PaO_2_) divided into deciles and referenced against the fourth decile (PaO_2_, 83 to 93). The adjusted model included the following covariates: fraction of inspired oxygen (deciles), Acute Physiology and Chronic Health Evaluation III (APACHE III) index of illness severity in which the oxygen component of the APACHE III scoring system was removed, year of admission, treatment limitation on admission to intensive care unit, patient's lowest glucose level in the first 24 hours, hospital characteristics, patient indigenous status and hospital source from home. 95% CI, 95% confidence interval.

When PaO_2 _was divided into deciles and modelled as a predictor of hospital mortality, it was statistically significant at a univariate level (*P *< 0.0001), but with only the lowest two deciles having ORs significantly greater than the norm (Figure 1). After adjustment for FiO_2 _and the covariates described in Additional file 1, Statistical appendix, Model cluster 2, PaO_2 _was no longer predictive of hospital mortality (*P *= 0.21), although those patients with isolated hypoxemia (PaO_2 _< 60 mmHg) had a significantly greater risk (OR 1.2 (95% CI, 1.0 to 1.5), *P *= 0.03) (Figure 1). Importantly, 492 patients (42.1%) with isolated hypoxemia were receiving deliberate decreases of FiO_2 _to <0.8 at the time of their hypoxemia. There was no statistical evidence that patients with higher PaO_2 _levels had significantly greater risk of hospital mortality.

When the corresponding time period used by the EMShockNet study group [6] (2001 to 2005) was considered, after adjustment (Additional file 1, Statistical appendix, Model cluster 2), oxygenation was not predictive of mortality (*P *= 0.16) (hyperoxia: OR 1.3 (95% CI, 0.9 to 1.8), *P *= 0.16; hypoxia: OR 1.3 (95% CI, 1.0 to 1.6), *P *= 0.06). When more detailed information was obtained from a random sample of 100 patients, the worst PaO_2 _value over the first 24 hours was significantly more representative of mean PaO_2 _than the first PaO_2 _value measured upon admission used by the EMShockNet study group [6]. This was true for the first 24 hours (Pearson's *r *= 0.70 versus Pearson's *r *= 0.50, *P *< 0.0001), the first 48 hours (Pearson's *r *= 0.63 versus Pearson's *r *= 0.38, *P *< 0.0001) and the first 72 hours (Pearson's *r *= 0.60 versus Pearson's *r *= 0.34, *P *< 0.0001).

## Discussion

### Key findings

We conducted a large, multicentre, cohort study of patients admitted to ICUs in ANZ after resuscitation from cardiac arrest to examine the relationship between hyperoxia and patient outcome. We initially found that hyperoxia was relatively uncommon and had only a weak relationship with risk of death. This relationship was significantly reduced by the addition of illness severity scores. In addition, once Cox proportional hazards modelling of survival, sensitivity analyses using deciles of hypoxemia, time period matching and defining hyperoxia in keeping with experimental studies [10-13] as PaO_2 _> 400 mmHg, hyperoxia had no independent association with mortality. Finally, after adjustment for FiO_2 _and relevant covariates, PaO_2 _was no longer predictive of hospital mortality. Thus, hyperoxia was relatively uncommon, and it had no robust and consistently reproducible independent relationship with mortality.

### Comparison with other studies

Until very recently, concerns about the possible risks associated with hyperoxia during and after recovery from cardiac arrest were based on animal experiments [10-13]. In this regard, several experimental studies have suggested that hyperoxia can increase oxidative stress [14], induce more severe histopathological changes [10] and worsen neurological injury [15]. On the other hand, two studies have failed to confirm such findings [16,17], and two other often-quoted major studies did not actually assess animals after cardiac arrest [10,12]. Nonetheless, despite the lack of human data, the International Liaison Committee on Resuscitation moved to advocate the avoidance of arterial hyperoxia. The committee instead advocated the targeting of arterial oxygen saturation not exceeding 94% to 96% [18]. In response to these issues, in June 2010, the EMShockNet investigators reported that, in a cohort of 6,326 USA patients who had survived nontraumatic cardiac arrest and were admitted to the ICU, hyperoxia was independently associated with increased risk (OR 1.6) of in-hospital mortality. This was the largest clinical study to date of the association between hyperoxia after cardiac arrest and mortality. Our findings should therefore be evaluated in direct comparison to the EMShockNet study and have been specifically configured to facilitate such comparison.

Several important observations emerge from such comparison. First, the baseline characteristics of the U.S. and ANZ patients appear almost identical, although no information was available on the APACHE scores for the USA cohort. Despite such similarities, there were striking differences in the lowest body temperatures recorded (ANZ 34.9°C, USA 36°C). These differences may reflect greater uptake of therapeutic hypothermia in ANZ and make the observations from our cohort more relevant to current recommended practice [4,5]. However, we cannot determine whether greater use of therapeutic hypothermia accounts for the difference in the proportion of survivors discharged to home (65% in ANZ as compared to 44% in the USA, approximately a 50% relative increase in favourable outcome).

In the ANZ cohort, hyperoxia occurred in only 10.6% of patients as compared with 18% in the USA, and mortality in the hyperoxic group was identical to that in the hypoxia/poor O_2 _transfer group, instead of being much greater. Importantly, the relationship between hyperoxia and in-hospital mortality appeared much weaker using the same modelling used by the EMShockNet investigators. Even more importantly, this relationship could not be confirmed when a different threshold for hyperoxia was applied which mimicked that reported in experimental studies [11-13] (rather than using a seemingly arbitrary cutoff point of 300 mmHg), when a Cox proportional hazards model was used, when PaO_2 _was split into deciles, when FiO_2 _was taken into account or when the same time period (2000 to 2005) was used for analysis. In the aggregate, these observations suggest that the relationship between hyperoxia and mortality is dependent on the individual healthcare system, the statistical model used, the time period examined and the definitions used. Such features are not consistent with a robust and reproducible biological phenomenon.

### Study significance

Our findings imply that it is incorrect and premature to conclude that hyperoxia is an independent risk factor for mortality in patients resuscitated from nontraumatic cardiac arrest. In particular, we contend that hyperoxia implies the administration of high FiO_2 _fractions, making it more likely for hyperoxia to actually be a marker of illness severity than a biological toxin. This notion is supported by the significant decrease in ORs for mortality once APACHE scores were added to the model and the disappearance of significant ORs once FiO_2 _was added to the model. Moreover, the definition of 'hypoxia' used by the EMShockNet investigators (reproduced here to facilitate comparison under the term 'hypoxia/poor O_2 _transfer') included patients with a P/F ratio <300, together with patients with PaO_2 _< 60 mmHg. This approach conflates physiologically relevant lack of oxygen at the tissue level (true hypoxia) with a gas transfer problem. When we examined isolated hypoxemia (PaO_2 _< 60 mmHg), we found that it was nearly as frequent as hyperoxia. The risks of cerebral injury associated with hypoxemia are well known [19-21], and hypoxemic patients in our cohort had particularly poor outcomes. Importantly, after adjustment for FiO_2 _and relevant covariates, PaO_2 _was no longer predictive of hospital mortality. These observations suggest that the association seen in the models that do not include FiO_2 _may simply reflect the fact that hyperoxia is an indirect marker of higher FiO_2 _(that is, the higher the FiO_2_, the greater the PaO_2_) and that a higher FiO_2 _is a marker of illness severity (that is, the sicker the patient is perceived to be, the greater the FiO_2 _administered in an emergency situation). However, the link between FiO_2 _and outcome is independent of APACHE score. Thus, FiO_2 _cannot be considered simply a marker of disease severity. The physician can lower PaO_2 _and FiO_2 _levels at the same time and avoid inducing hyperoxia. Only interventional studies can clarify whether the association between oxygenation and outcome is truly a causal relationship.

All the above observations have potential clinical relevance. For example, emergency responders may not have access to pulse oximetry or blood gas analysis, or such techniques may be unreliable immediately following cardiac arrest because of decreased peripheral perfusion. If fear of hyperoxia led emergency responders, in the absence of adequate monitoring, to limit FiO_2 _levels, logically more people would likely be exposed to the risk of hypoxemia. In our study, >40% of patients with hypoxemia might have had correction of their hypoxemia had a higher FiO_2 _level been induced. Thus, if concerns about the alleged ill effects of hyperoxia or high FiO_2 _administration were taken into the clinical arena to avoid a condition whose association with mortality is uncertain, more patients might be exposed to a condition whose adverse cerebral effects are well established. Given our findings, we counsel against implementing policies of deliberately induced decreases in FiO_2 _unless accurate continuous pulse oximetry monitoring is in place. Importantly, in no way do we advocate, promote or justify hyperoxia in this setting. However, lowering FiO_2 _is justified only if good transcutaneous or arterial oxygenation monitoring is available.

### Strengths and limitations

This study has several strengths. It involved more than 12,000 patients from 125 ICUs in two countries, making it the largest study of its type conducted so far and making its findings reflective of all ICUs in ANZ [22,23]. It included a multifaceted assessment of the independent relationship between hyperoxia and outcome using multiple models and adjusting for illness severity. However, like other studies of association using a large database, it is limited by the nature of the data available and by the fact that no causal inferences can be drawn. The assessment of oxygenation status in the first 24 hours was based on the 'worst' possible arterial blood gas result, while the EMShockNet study used the 'first' ICU arterial blood gas measurement for evaluation. Thus, patients may have been exposed to hyperoxia and may not have been identified in our study. However, using a random sample of 100 patients, we found that the measurement used in our study was more closely representative of overall mean oxygenation status in ICU patients during the first 24 to 48 hours after admission (when reperfusion injury occurs) than the first set of blood gas measurements obtained in the ICU. In our study, data were missing for only 5.4% of patients compared with 27.6% in the EM ShockNet study, making selection bias in our study less likely. One-third of patients had a lowest body temperature <34°C. Clinical knowledge (confirmed by the EMShockNet data) that such severe spontaneous hypothermia is uncommon suggests that many patients were therefore treated with induced hypothermia as is common in ANZ [24-28]. This finding distinguishes our study from the US investigation because it is in keeping with current recommendations. Unfortunately, however, our database does not enable us to identify which patients had induced versus spontaneous hypothermia. Finally, we are unable to comment on the causes of death or consider other potential confounding variables that were not collected as part of the ANZICS-APD.

### Future studies

More investigations appear necessary, perhaps using other national databases [29]. Prospective investigations with focused data collection are also needed. If such studies confirmed a postive association, interventional strategies should be tested; if not, interventional studies would not seem justified.

## Conclusions

In a large, multicentre, cohort study of patients admitted to the ICU after resuscitation from cardiac arrest, we found that hyperoxia was relatively uncommon. On the basis of initial multivariable analysis, it had only a weak independent relationship with mortality. This relationship could not be confirmed on the basis of sensitivity analysis, adjusted Cox proportional hazards modelling, after taking FiO_2 _into account or after adjusting for time period, making it unlikely that it represents a reproducible biological phenomenon. Our findings support arguments against implementing policies of deliberate decreases in FiO_2 _unless accurate and reliable pulse oximetry monitoring is available.

## Key messages

• When the worst set of arterial blood gases is used for assessment, hyperoxia is uncommon in the first 24 hours after ICU admission in patients resuscitated from cardiac arrest.

• Using the same approach, isolated hypoxemia is just as common.

• Hyperoxia in these patients has a weak, model-dependent and nonreproducible association with mortality.

• Unless accurate and reliable pulse oximetry is available to prevent hypoxemia, a policy of reducing FiO_2 _to avoid possible hyperoxia is not justified and may not be prudent.

## Abbreviations

ANZ: Australia and New Zealand; ANZICS: Australian and New Zealand Intensive Care Society; APACHE III: Acute Physiology and Chronic Health Evaluation III; CORE: Centre for Outcomes and Resource Evaluation; FiO_2_: inspired fraction of oxygen; PaO_2_: arterial oxygen tension.

## Competing interests

The authors declare that they have no competing interests.

## Authors' contributions

RB conceived the study in conjunction with the other authors and wrote the initial draft of the manuscript. MB conceived the study in conjunction with the other authors and performed the statistical analysis. GME conceived the study in conjunction with the other authors and reviewed and modified the final manuscript. AN conceived the study in conjunction with the other authors and reviewed and modified the final manuscript. DP conceived the study in conjunction with the other authors and reviewed and modified the final manuscript. GKH conceived the study in conjunction with the other authors and reviewed and modified the final manuscript. MCR conceived the study in conjunction with the other authors and reviewed and modified the final manuscript. ME assisted with the study and obtained and provided information on arterial blood gases in a selected cohort of cardiac arrest patients. DJC conceived the study in conjunction with the other authors and reviewed and modified the final manuscript.

## Supplementary Material

Additional file 1 **Statistical appendix with details of multiple statistical models linking oxygen status with outcome**. Click here for file

## References

[B1] Peberdy MA Kaye W Ornato JP Larkin GL Nadkarni V Mancini ME Berg RA Nichol G Lane-Trultt T Cardiopulmonary resuscitation of adults in the hospital: a report of 14720 cardiac arrests from the National Registry of Cardiopulmonary Resuscitation Resuscitation 2003 58 297 308 10.1016/S0300-9572(03)00215-6 12969608

[B2] Stiell IG Wells GA Field B Spaite DW Nesbitt LP De Maio VJ Nichol G Cousineau D Blackburn J Munkley D Luinstra-Toohey L Campeau T Dagnone E Lyver M Ontario Prehospital Advanced Life Support Study Group Advanced cardiac life support in out-of-hospital cardiac arrest N Engl J Med 2004 351 647 656 10.1056/NEJMoa040325 15306666

[B3] Negovsky VA The second step in resuscitation: the treatment of the 'post-resuscitation disease' Resuscitation 1972 1 1 7 10.1016/0300-9572(72)90058-5 4653025

[B4] Bernard SA Gray TW Buist MD Jones BM Silvester W Gutteridge G Smith K Treatment of comatose survivors of out-of-hospital cardiac arrest with induced hypothermia N Engl J Med 2002 346 557 563 10.1056/NEJMoa003289 11856794

[B5] Hypothermia after Cardiac Arrest Study Group Mild therapeutic hypothermia to improve the neurologic outcome after cardiac arrest N Engl J Med 2002 346 549 563 10.1056/NEJMoa012689 11856793

[B6] Kilgannon JH Jones AE Shapiro NI Angelos MG Milcarek B Hunter K Parrillo JE Trzeciak S Emergency Medicine Shock Research Network (EMShockNet) Investigators Association between arterial hyperoxia following resuscitation from cardiac arrest and in-hospital mortality JAMA 2010 303 2165 2171 10.1001/jama.2010.707 20516417

[B7] Stow PJ Hart GK Higlett T George C Herkes R McWilliam D Bellomo R for the ANZICS Database Management Committee Development and implementation of a high-quality clinical database: the Australian and New Zealand Intensive Care Society Adult Patient Database J Crit Care 2006 21 133 141 10.1016/j.jcrc.2005.11.010 16769456

[B8] Knaus WA Wagner DP Draper EA Zimmerman JE Bergner M Bastos PG Sirio CA Murphy DJ Lotring T Damiano A The APACHE III prognostic system. Risk prediction of hospital mortality for critically ill hospitalized adults Chest 1991 100 1619 1636 10.1378/chest.100.6.1619 1959406

[B9] D'Agostino RB Jr Propensity scores in cardiovascular research Circulation 2007 115 2340 2343 10.1161/CIRCULATIONAHA.105.594952 17470708

[B10] Douzinas EE Patsouris E Kypriades EM Makris DJ Andrianakis I Korkolopoulou P Boursinos V Papalois A Sotiropoulou C Davaris P Roussos C Hypoxaemic reperfusion ameliorates the histopathological changes in the pig brain after a severe global cerebral ischaemic insult Intensive Care Med 2001 27 905 910 10.1007/s001340100932 11430548

[B11] Balan IS Fiskum G Hazelton J Cotto-Cumba C Rosenthal RE Oximetry-guided reoxygenation improves neurological outcome after experimental cardiac arrest Stroke 2006 37 3008 3013 10.1161/01.STR.0000248455.73785.b1 17068310 PMC2600845

[B12] Douzinas EE Andrianakis I Pitaridis MT Karmpaliotis DJ Kypriades EM Betsou A Gratsias Y Sotiropoulou C Papalois A Roussos C The effect of hypoxemic reperfusion on cerebral protection after a severe global ischemic insult Intensive Care Med 2001 27 269 275 10.1007/s001340000796 11280647

[B13] Richards EM Fiskum G Rosenthal RE Hopkins I McKenna MC Hyperoxic reperfusion after global ischemia decreases hippocampal energy metabolism Stroke 2007 38 1578 1584 10.1161/STROKEAHA.106.473967 17413048 PMC2601708

[B14] Liu Y Rosenthal RE Haywood Y Miljkovic-Lolic M Vanderhoek JY Fiskum G Normoxic ventilation after cardiac arrest reduces oxidation of brain lipids and improves neurological outcome Stroke 1998 29 1679 1686 10.1161/01.STR.29.8.1679 9707212

[B15] Zwermer CF Whitesall SE D'Alecy LG Cardiopulmonary-cerebral resuscitation with 100% oxygen exacerbates neurological dysfunction following nine minutes of normothermic cardiac arrest in dogs Resuscitation 1994 27 159 170 10.1016/0300-9572(94)90009-4 8086011

[B16] Zwermer CF Whitesall SE D'Alecy LG Hypoxic cardiopulmonary-cerebral resuscitaiton fails to improve neurological outcome following cardiac arrest in dogs Resuscitation 1995 29 225 236 10.1016/0300-9572(94)00848-A 7667554

[B17] Lipinski CA Hicks SD Callaway CW Normoxic ventilation during resuscitation and outcome from asphyxial cardiac arrest in rats Resuscitation 1999 42 221 229 10.1016/S0300-9572(99)00083-0 10625163

[B18] Neumar RW Nolan JP Adrie C Aibiki M Berg RA Böttiger BW Callaway C Clark RS Geocadin RG Jauch EC Kern KB Laurent I Longstreth WT Jr Merchant RM Morley P Morrison LJ Nadkarni V Peberdy MA Rivers EP Rodriguez-Nunez A Sellke FW Spaulding C Sunde K Vanden Hoek T Post-cardiac arrest syndrome: epidemiology, pathophysiology, treatment, and prognostication. A consensus statement from the International Liaison Committee on Resuscitation (American Heart Association, Australian and New Zealand Council on Resuscitation, European Resuscitation Council, Heart and Stroke Foundation of Canada, InterAmerican Heart Foundation, Resuscitation Council of Asia, and the Resuscitation Council of Southern Africa); the American Heart Association Emergency Cardiovascular Care Committee; the Council on Cardiovascular Surgery and Anesthesia; the Council on Cardiopulmonary, Perioperative, and Critical Care; the Council on Clinical Cardiology; and the Stroke Council Circulation 2008 118 2452 2483 10.1161/CIRCULATIONAHA.108.190652 18948368

[B19] Tsui SS Schultz JM Shen I Ungerleider RM Postoperative hypoxemia exacerbates potential brain injury after deep hypothermic circulatory arrest Ann Thorac Surg 2004 78 188 196 10.1016/j.athoracsur.2003.11.048 15223426

[B20] Martin LJ Brambrink AM Lehmann C Portera-Cailliau C Koehler R Rothstein J Traystman RJ Hypoxia-ischemia causes abnormalities in glutamate transporters and death of astroglia and neurons in newborn striatum Ann Neurol 1997 42 335 348 10.1002/ana.410420310 9307255

[B21] Stahel PF Smith WR Moore EE Hypoxia and hypotension, the "lethal duo" in traumatic brain injury: implications for prehospital care Intensive Care Med 2008 34 402 404 10.1007/s00134-007-0889-3 17938886

[B22] ANZIC Influenza Investigators Critical care services and 2009 H1N1 influenza in Australia and New Zealand N Engl J Med 2009 361 1925 1934 10.1056/NEJMoa0908481 19815860

[B23] Webb SA Seppelt IM ANZIC Influenza Investigators Pandemic (H1N1) 2009 influenza ("swine flu") in Australian and New Zealand intensive care Crit Care Resusc 2009 11 170 172 19737115

[B24] Whitfield AM Coote S Ernest D Induced hypothermia after out of hospital cardiac arrest: one hospital's experience Crit Care Resusc 2009 11 97 100 19485872

[B25] Jones DA Management of cardiac arrest patients in the ICU: is keeping a cool head the standard of care? Crit Care Resusc 2009 11 91 93 19485870

[B26] Moran JL Peake SL Solomon P Hypothermia as therapy in cerebral injury Crit Care Resusc 2002 4 86 92 16573410

[B27] Bernard SA Hypothermia improves outcome from cardiac arrest Crit Care Resusc 2005 7 325 327 16539589

[B28] Bernard SA Smith K Cameron P Masci K Taylor DM Cooper DJ Kelly AM Silvester W Rapid Infusion of Cold Hartmanns (RICH) Investigators Induction of therapeutic hypothermia by paramedics after resuscitation from out-of-hospital ventricular fibrillation cardiac arrest: a randomized controlled trial Circulation 2010 122 737 742 10.1161/CIRCULATIONAHA.109.906859 20679551

[B29] Harrison DA Rowan KM Outcome prediction in critical care: the ICNARC model Curr Opin Crit Care 2008 14 506 512 10.1097/MCC.0b013e328310165a 18787441

[B30] Halpern NA Pastores SM Thaler HT Greenstein RJ Changes in critical care beds and occupancy in the United States 1985-2000: Differences attributable to hospital size Crit Care Med 2006 34 2105 2112 10.1097/01.CCM.0000227174.30337.3E 16755256

