# Supplementary material for: Arterial hyperoxia and in-hospital mortality after resuscitation from cardiac arrest
Source: Crit Care. 2011 Mar 8;15(2):R90. doi: 10.1186/cc10090 (PMC3219350; doi:10.1186/cc10090)
Supplement: Additional file 1 — Statistical appendix with details of multiple statistical models linking oxygen status with outcome. [file cc10090-S1.DOCX]

**STATISTICAL APPENDIX**

**eTable 1.** Sensitivity Analysis Incorporating a Propensity Score for Hyperoxia Exposure into the Multiple Logistic Regression Model

| **Variable** | **Hospital Mortality**  **OR (95%CI)** | **P-value** |
| --- | --- | --- |
| Propensity score for hyperoxia exposure | 0.9 (0.9- 0.9) | <0.0001 |
| AP3no-ox ^a^ | 1.5 (1.5-1.6) | <0.0001 |
| Treatment Limitation ^b^ | 5.1 (3.7-7.0) | <0.0001 |
| Year of admission | 0.9 (0.9-0.9) | <0.0001 |
| Lowest Glucose in the first 24 hours | 1.1 (1.0-1.1) | <0.0001 |
| Hospital admission from home | 1.3 (1.1-1.4) | 0.0002 |
| Hypoxia vs Normoxia | 1.2 (1.1-1.4) | 0.006 |
| Hyperoxia vs Normoxia | 1.2 (1.0-1.5) | 0.04 |

Indigenous status was removed from the model for non-significance (p<0.01): (OR 1.3 (1.0-1.8) p=0.04),

^a^ APACHEIII-j risk of death with the oxygen component removed from the calculation algorithm. OR represents the increased risk associated with a 10% increase in the calculated risk of death

^b^ A treatment limitation order or palliative care has been coded for the patient

**eTable 2: Comparison of Mortality for all variables**

| **Variable** | **Alive (5140)** | **Deceased (6968)** | **P-value**^a^ |
| --- | --- | --- | --- |
| Age | 64.3 (52-74.2) | 68.9 (56.15-77.8) | <0.0001 |
| Acute Renal Failure | 9% (458) | 27% (1910) | <0.0001 |
| Cancer | 2% (115) | 4% (298) | <0.0001 |
| Chronic condition |  |  |  |
| Cardiovascular | 18% (925) | 21% (1470) | <0.0001 |
| Liver | 1% (49) | 2% (145) | <0.0001 |
| Renal Failure | 4% (221) | 6% (447) | <0.0001 |
| Respiratory | 7% (360) | 10% (684) | <0.0001 |
| Cirrhosis | 1% (49) | 2% (146) | <0.0001 |
| Lowest Glucose | 6.38 (2.97) | 7.21 (4.37) | <0.0001 |
| Hepatic failure | 0% (9) | 1% (61) | <0.0001 |
| Hospital Admission Source |  |  |  |
| Chronic Care | 1% (76) | 2% (112) | 0.57 |
| Home | 64% (3310) | 70% (4865) | <0.0001 |
| Other Hospital | 25% (1305) | 22% (1542) | <0.0001 |
| Other ICU | 2% (104) | 2% (127) | 0.42 |
| Length of Stay in hosp (hrs) | 358 (197.3-654) | 81.38 (35.95-194.17) | <0.0001 |
| Hospital Type |  |  |  |
| Metropolitan | 18% (947) | 25% (1723) | <0.0001 |
| Private | 8% (416) | 5% (371) | <0.0001 |
| Rural | 10% (500) | 11% (779) | 0.01 |
| Tertiary | 64% (3277) | 59% (4095) | <0.0001 |
| Heart Rate /min | 90 (55-116) | 106 (59-128) | <0.0001 |
| Highest heart Rate | 102 (90-120) | 110 (92-130) | <0.0001 |
| Hyperoxia (pao2 >400) | 5% (280) | 5% (345) | 0.22 |
| Hyperoxia (pao2 >300) | 10% (531) | 11% (754) | 0.39 |
| Hypoxia | 70% (3601) | 76% (5303) | <0.0001 |
| ICU admission Source |  |  |  |
| Emergency | 44% (2248) | 50% (3508) | <0.0001 |
| Operating Theatre | 16% (825) | 6% (436) | <0.0001 |
| Other Hospital | 18% (924) | 15% (1034) | <0.0001 |
| Ward | 22% (1131) | 28% (1982) | <0.0001 |
| Length of Stay in ICU (hours) | 85.5 (45.31-166.5) | 46.5 (16.5-95.1) | <0.0001 |
| Immunosuppressed by disease or therapy | 2% (128) | 5% (314) | <0.0001 |
| Indigenous | 5% (230) | 4% (285) | 0.21 |
| Intubated | 98% (5042) | 99% (6883) | 0.002 |
| Male gender | 67% (3459) | 62% (4343) | <0.0001 |
| Lowest Mean Arterial Pressure | 65.82 (12.42) | 59.67 (17.8) | <0.0001 |
| APACHEIII Risk of Death | 41.81 (28.42) | 71.65 (23.05) | <0.0001 |
| APACHEIII Risk of Death (no oxygen) | 35.99 (27.14) | 65.7 (24.47) | <0.0001 |
| Highest Respiratory Rate /min | 20.68 (7.47) | 22.91 (9.91) | <0.0001 |
| Systolic Blood Pressure Under 90 | 36% (1852) | 53% (3709) | <0.0001 |
| Lowest Systolic BP (mm Hg) | 94.39 (19.15) | 84.19 (27.93) | <0.0001 |
| Highest Temp | 37.2 (1.19) | 37.04 (1.62) | <0.0001 |
| Lowest Temp | 35.5 (34-36.2) | 35 (33.5-36) | <0.0001 |
| Treatment Limitation | 1% (72) | 7% (490) | <0.0001 |
| Year | 2005.93 (2.65) | 2005.67 (2.73) | <0.0001 |

^a^ Group comparisons are made using chi-square tests for equal proportion, student t-tests for normally distributed data and Wilcoxon Rank Sum otherwise

**Statistical appendix**

**Model 1**

An initial model for mortality was constructed in accordance with the EMShockNet^8^ which included age (deciles), gender, indigenous status, hospital location and ICU source prior to admission, acute renal failure, hypotension (systolic blood pressure <90 mm Hg), high heart rate (above median), gender, chronic co-morbidities (respiratory, renal, cardiovascular, liver), hepatic failure, immune suppression, cancer, cirrhosis and oxygenation level (hypoxia, normoxia, hyperoxia). To account for potential heterogeneity, hospital was also included as a covariate in the multivariate models. The predictive effects on in-hospital mortality were assessed for hyperoxia and hypoxia with results contrasted against normoxia. Goodness and quality of fit were determined using the Hosmer and Lemeshow goodness-of-fit test and the c-statistic for Area under the Receiver Operator Characteristic curve (AUC).

**Model cluster 2**

To improve discriminatory power, a second model was constructed with the inclusion of AP3no-ox as a marker of patient severity. To avoid any collinearity with AP3no-ox, all variables that in some way contributed to the calculation of AP3no-ox were removed. Additional covariates, independent of AP3no-ox but known to be predictors of mortality were identified a priori by investigators. These additional covariates included year of admission, treatment limitations, patient lowest glucose level in the first 24 hours, patient indigenous status, and hospital source from home. Both year of admission and glucose level (divided in quintiles) were initially treated as categorical variables and then as continuous variables where evidence of linearity was shown to exist.

**Model cluster 3**

To ensure that hyperoxia effects were not due to each patient’s propensity to be hyperoxic, a sensitivity analysis was conducted using the previous model with the addition of each patient’s propensity to be exposed to hyperoxia. Propensity scores were calculated in accordance with D’Agostino^9^ with all previously mentioned variables considered for inclusion.

To establish if oxygenation status was related to patient survival time, a Cox-proportional hazards regression model was constructed including hyperoxia, hypoxia, AP3-no-ox, year of admission, treatment limitation on admission to ICU, patient lowest glucose level in the first 24 hours, patient indigenous status, and hospital source from home.

To establish if the relationship between mortality and hyperoxia was dependent upon the choice of cut-off value used to define hyperoxia, two additional cut-offs (200 and 400) were considered. To further explore the relationship between PaO_2_ and mortality, PaO_2_ was divided into deciles and modelled against mortality using both univariate and multivariate analysis incorporating the previously described covariates as well as FiO_2_ divided into deciles.

To establish if the relationship between hyperoxia and mortality was time dependent, a final subgroup analysis was performed looking specifically at the time period 2001-2005.

To determine whether the worst value in the first 24 hours was more or less representative of mean PaO_2_ in the first 24, 48 or 72 hours than the first observation taken on admission, Pearson correlations were considered for a subset of 100 patients in whom all blood gas values were obtainable.

With 1,285 hyperoxia patients and 1,919 normoxia patients, this study had a 90% power with a two-sided p-value of 0.01 to detect a difference in proportion of 7%.
